# Acupuncture for insomnia after stroke: a systematic review and meta-analysis

**DOI:** 10.1186/s12906-016-1220-z

**Published:** 2016-07-19

**Authors:** Sook-Hyun Lee, Sung Min Lim

**Affiliations:** Department of Clinical Research on Rehabilitation, Korea National Rehabilitation Research Institute, 58 Samgaksan-ro, Gangbuk-gu, Seoul, 142-070 Republic of Korea

**Keywords:** Acupuncture, Intradermal acupuncture, Stroke, Insomnia, Review

## Abstract

**Background:**

Insomnia is the common complaint among patients with stroke. Acupuncture has increasingly been used for insomnia relief after stroke.

The aim of the present study was to summarize and evaluate evidence on the effectiveness of acupuncture in relieving insomnia after stroke.

**Methods:**

Seven databases were searched from inception through October 2014 without language restrictions. Randomized controlled trials (RCTs) were included if acupuncture was compared to placebo or other conventional therapy for treatment of insomnia after stroke. Assessments were performed using the Pittsburgh sleep quality index (PSQI), the insomnia severity index (ISI), the Athens insomnia scale (AIS), and the efficacy standards of Chinese medicine.

**Results:**

A total of 165 studies were identified; 13 RCTs met our inclusion criteria. Meta-analysis showed that acupuncture appeared to be more effective than drugs for treatment of insomnia after stroke, as assessed by the PSQI (weighted mean difference, 4.31; 95 % confidence interval [CI], 1.67–6.95; *P* = 0.001) and by the efficacy standards of Chinese medicine (risk ratio, 1.25; 95 % CI, 1.12–1.40; *P* < 0.001). Intradermal acupuncture had significant effects compared with sham acupuncture, as assessed by the ISI (weighted mean difference, 4.44; 95 % CI, 2.75–6.13; *P* < 0.001) and the AIS (weighted mean difference, 3.64; 95 % CI, 2.28–5.00; *P* < 0.001).

**Conclusions:**

Our results suggest that acupuncture could be effective for treating insomnia after stroke. However, further studies are needed to confirm the role of acupuncture in the treatment of this disorder.

**Electronic supplementary material:**

The online version of this article (doi:10.1186/s12906-016-1220-z) contains supplementary material, which is available to authorized users.

## Background

Stroke is the second-leading global cause of death behind heart disease, accounting for 11.13 % of total deaths worldwide [1]. In addition, survivors often suffer from not only pain and various physical disabilities but also mood disorders such as depression [2]. Such physical and emotional consequences of stroke could have multiple effects on a patient’s sleeping pattern. Previous studies have reported that the sleep-wake cycle is frequently disturbed after stroke [3, 4].

Insomnia is the most common sleep complaint, affecting approximately 40–60 % of stroke patients [3]. This frequency is higher than what observed in patients without a stroke (10–40 %) [5]. Insomnia after stroke is caused mainly by anxiety resulting from hyperactivity of the sympathetic nervous system [3–6]. In addition, post-stroke insomnia might be affected by damaged brain lesions resulted from the stroke, age, degree of disability after stroke, anxiety disorder, antipsychotic drugs, depression, and other comorbidities [6, 7]. During stroke recovery, psychological stress due to insomnia affects the effectiveness of therapy and the prognosis; it also affects quality of life, mental health, and rehabilitation [8–10].

Although effective pharmacological treatments are available, significant side effects have limited their clinical applications and long-term use [10]. Of the complementary treatment modalities, acupuncture has been one of the most popular and safest [11].

Acupuncture has been widely used to treat a variety of clinical conditions, particularly those involving pathological changes in neuroendocrinology, such as menopause, depression, and insomnia [12]. Acupuncture is able to regulate the functioning of the heart and brain through stimulation of certain acupoints on the body. Many published clinical studies, including randomized controlled trials (RCTs), have explored acupuncture as a treatment for insomnia. Most reports have demonstrated positive clinical effects of acupuncture in the treatment of insomnia. Acupuncture treatment has also been reported to reduce sleep onset latency and increase sleep duration and sleep efficiency [13].

A few recent systematic reviews have examined the effectiveness of acupuncture in the treatment of insomnia [12, 14, 15]. However, none has focused on insomnia after stroke. Furthermore, the effectiveness of acupuncture in treating insomnia after stroke has not been fully determined. The aim of the present study was to summarize and evaluate evidence on the effectiveness of acupuncture for insomnia relief after stroke.

## Methods

### Search methods for identification of studies

The search was performed without restriction to language or year of publication. We searched Medline, EMBASE, and the Cochrane Central Register of Controlled Trials from database inception through October 2014. For Korean publications, we searched three Korean medical databases (Research Information Service System, National Discovery for Science Leaders, and OASIS). For Chinese articles, we searched the China National Knowledge Infrastructure (CNKI). The keywords used for the search were “stroke OR apoplexy OR cva OR cerebrovascular attack OR cerebrovascular accident OR cerebral infarction OR cerebral hemorrhage” AND “acupuncture OR acupoints OR electroacupuncture OR electro-acupuncture OR auriculotherapy OR auriculoacupuncture” AND “insomnia” in each database language. The search strategy was adjusted for each database (Appendix).

### Inclusion/exclusion criteria

Relevant clinical trials were included if the following criteria were met: 1) they were randomized, controlled trials (RCTs); 2) they included patients diagnosed with insomnia after stroke; 3) stroke patients with insomnia at baseline were enrolled, and 4) they studied insomnia as an outcome measure. Trials were excluded if the study design did not allow evaluation of the effects of acupuncture on insomnia after stroke; that is, studies were excluded if they 1) compared different types of acupuncture, 2) adopted complex treatment without examining the effects of acupuncture alone, or 3) reported insufficient information.

### Data extraction

Two reviewers (L.S.H. and L.S.M.) independently extracted data using a standardized data extraction form and reached consensus on all items. Extracted data included authors, year of publication, sample size, interventions, main outcomes, and adverse events.

Instruments of the outcome measurements that were reported in the included studies were the Pittsburgh sleep quality index (PSQI), the efficacy standards of Chinese medicine, the Insomnia Severity Index (ISI), and the Athens insomnia scale (AIS).

The PSQI consists of 19 self-rated questions, which are grouped into seven component scores ranging from 0 to 3 each. The seven component scores are then summed to yield a global PSQI score, which has a range of 0 to 21, with higher scores indicating worse sleep quality. Specifically, a score of “0” indicates no difficulty, whereas a score of “21” indicates severe difficulties in all areas [16]. The ISI is a brief self-report instrument measuring a patient’s perception of insomnia. The seven items are rated on a 0-to-4 scale, and the total score ranges from 0 to 28. A higher score indicates more severe insomnia [17]. The AIS is a self-administered psychometric instrument consisting of eight items. Each item of the AIS can be rated from 0 to 3 for a total score range of 0–24, with a score of “0” indicating no problem at all and a score of “24” indicating very serious problems in all areas [18]. We extracted data on the mean change from baseline measures (the “Mean”, Fig. 3). The standard deviation of changes from baseline was determined using a correlation coefficient from a previously published study [19].

The efficacy standards of Chinese medicine is a measurement tool for the assessment of the states of the patients with complete improvement (recovery of normal sleep duration), partial improvement (increased sleep duration more than three hours), and no improvement after treatment [12]. Response rate was calculated based on proportion of the effective (complete or partial improvement) and not effective (no improvement) patients. We also considered measures of general safety reported for acupuncture as a treatment. We extracted data on the number of participants with improvement as an “Event” (Fig. 3).

### Quality assessment

The two reviewers independently assessed the methodological quality and the risk of bias of the included studies by means of the risk of bias (ROB) tool in the Cochrane Handbook for Systematic Reviews of Interventions (Version 5.0.2). This instrument consists of 8 domains: random sequence generation; allocation concealment; blinding of patients, personnel, and outcome assessors; incomplete outcome data; selective outcome reporting; and other sources of bias. The tool ranks evidence from research studies as having “high,” “low,” or “unclear” levels of bias; it is also appropriate for evaluating the methodological quality of RCTs. In cases in which the reviewers’ opinions differed, a joint opinion was reached through discussion.

### Statistical analysis

All statistical analyses were performed with Reviewer Manager Software, version 5.3 (Cochrane Collaboration, Oxford, UK). Summary estimates of treatment effects were calculated using a random-effects model. The impact of acupuncture on dichotomous data was expressed as the risk ratio (RR); for continuous outcomes, the mean difference was calculated with a 95 % confidence interval (CI). The statistical heterogeneity in the subgroups was analyzed using the *I*^*2*^ test and was considered to be significant when *I*^*2*^ was greater than 50 %. Even when a low heterogeneity was detected, a random-effects model was applied, because the validity of tests of heterogeneity can be limited with a small number of component studies. Publication bias was detected using a funnel plot.

## Results

### Study description

**Fig. 1:**
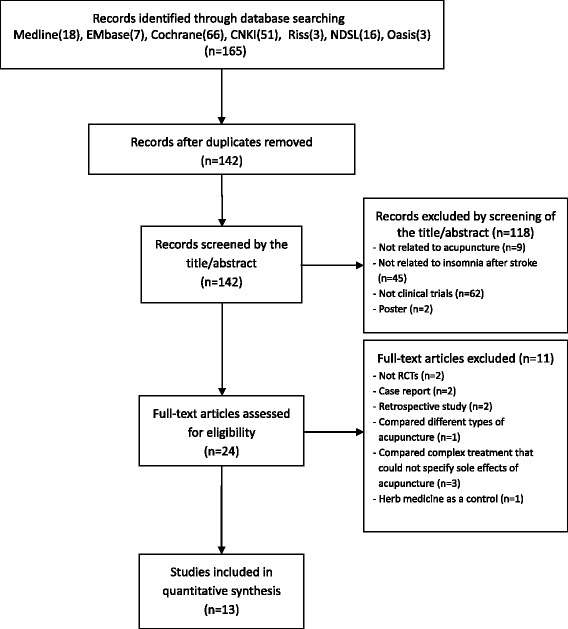
Flow chart of the trial selection process

We identified 165 publications; 13 met the eligibility criteria (Fig. 1). The articles included in the analysis are summarized in Table 1. The 13 articles were published from 2004 to 2012. Two originated in Korea [20, 21] and 11 were from China [8, 22–31]. The language of publication was English [20, 21] or Chinese [8, 22–31].

**Table 1:** Summary of randomized controlled trials of acupuncture for insomnia after stroke

Author (year) Country	Sample Size	Participants				Intervention Group Regimen	Control group regimen	Main outcomes	Results
		Average age (years)	Sex (male/female)	Severity of insomnia	Time since stroke				
Ye 2013 [22]	85	(a) 62.8 ± 7.2 (b) 67.3 ± 8.3	(a) 23/20 (b) 20/22	(a) n.r. (b) n.r.	(a) 15.9 ± 3.5 days (b) 14.17 ± 2.9 days	(a) AT (n = 43) (GV-20, EX-HN3, EX-HN1, HT-7, EX/ 5 times a week for 4 weeks, 30 min)	(b) Drugs (n = 42) (Alprazolam 0.4 mg once a day for 4 weeks)	(1) PSQI (2) Efficacy standards of Chinese medicine	(1) Significant differences in PSQI scores (*P* < 0.05) (2) Significant differences in Effective rates (*P* < 0.01)
Li 2012 [23]	300	(a) 49.2 (b) 51.3	(a) 79/71 (b) 77/73	(a) n.r. (b) n.r.	(a) n.r. (b) n.r.	(a) AT + Drugs (n = 150) (BL-62, KI-6, HT-7/ once a day for 10 days, 30 min)	(b) Drugs (n = 150) (Estazolam 0.5 mg once a day for 10 days)	(1) Efficacy standards of Chinese medicine	(1) Significant differences in Effective rates (*P* < 0.05)
Huang 2012 [24]	84	(a) n.r. (b) n.r.	(a) n.r. (b) n.r.	(a) n.r. (b) n.r.	(a) n.r. (b) n.r.	(a) AT (n = 42) (GV-20, EX-HN1, Auricular Shenmen, EX-HN3, EX, KI-6, BL-62/ 6 times a week for 2 weeks, 30 min)	(b) Drugs (n = 42) (Estazolam 2 mg 4–7 times a day for 2 weeks)	(1) Efficacy standards of Chinese medicine	(1) Significant differences in Effective rates (*P* < 0.05)
Wu 2012 [25]	80	(a) 67.6 ± 10.4 (b) 66.2 ± 9.6	(a) 20/20 (b) 18/22	(a) n.r. (b) n.r.	(a) n.r. (b) n.r.	(a) AA (n = 40) (Auricular Shenmen, heart, kidney, subcortex, internal ear/ 4–5 times a day for 10 days)	(b) Drugs (n = 40) (Estazolam 2 mg once a day for 10 days)	(1) Efficacy standards of Chinese medicine	(1) Significant differences in Effective rates (*P* < 0.01)
Huang 2011 [26]	60	(a) 66 (b) 67	(a) 11/19 (b) 13/17	(a) AIS > 6 (b) AIS > 6	(a) n.r. (b) n.r.	(a) AT + Drugs (n = 30) (GV-20, EX-HN1, SP-6, BL-23, HT-7, EX/ once a day for 14 days, 30 min)	(b) Drugs (n = 30) (Bailemianjiaonang 0.27 g × 4 capsules twice a day for 20 days)	(1) PSQI	(1) Significant differences in PSQI scores (*P* < 0.01)
Sun 2011 [27]	60	(a) 40 ± 15 (b) 40 ± 15	(a) 14/16 (b) 15/15	(a) PSQI 13.31 ± 2.4 (b) PSQI 12.09 ± 3.1	(a) 4.0 ± 2.6 years (b) 3.9 ± 2.8 years	(a) AT + AA (n = 30) (**AT**: GV-20, HT-7, SP-6, GB-20, EX-HN5, EX-HN1, EX/ once a day for 20 days, 30 min); **AA**: HT-7, sympathetic/ twice a day for 20 days, 15 ~ 20 min)	(b) Drugs (n = 30) (Estazolam 2 mg once a day for 20 days)	(1) PSQI (2) Efficacy standards of Chinese medicine	(1) Significant differences in PSQI scores (*P* < 0.05) (2) Significant differences in Effective rates (*P* < 0.05)
Ye 2010 [28]	60	(a) 61.5 ± 3.7 (b) 62.4 ± 4.9	(a) 16/14 (b) 17/13	(a) n.r. (b) n.r.	(a) n.r. (b) n.r.	(a) AT (n = 30) (GV-24, GV-20, GV-16, GV-11, EX-HN1/ 6 times a week for 4 weeks, 30 min)	(b) Drugs (n = 30) (Diazepam 5.0 mg once a day for 4 weeks)	(1) Efficacy standards of Chinese medicine	(1) Significant differences in Effective rates (*P* < 0.01)
Lee 2009 [20]	52	(a) 66.7 ± 11.0 (b) 66.0 ± 9.6	(a) 12/15 (b) 12/13	(a) ISI 18.4 ± 2.7, AIS 15.8 ± 2.4 (b) ISI 18.1 ± 2.6, AIS 14.9 ± 2.2	(a) n.r. (b) n.r.	(a) IA (n = 27) (He7, EH6/ once a day for 3 days)	(b) Sham AT (n = 25)	(1) ISI (2) AIS	(1)Significant differences in ISI scores (*P* < 0.01) (2)Significant differences in AIS scores (*P* < 0.01)
Lu 2008 [29]	50	(a) 61.48 ± 3.72 (b) 62.40 ± 4.88	(a) 14/11 (b) 15/10	(a) PSQI 16.64 ± 2.3 (b) PSQI 17.28 ± 2	(a) 0.3 ~ 1 years (b) 0.3 ~ 1 years	(a) AT (n = 25) (GV-24, GV-20, GV-16, GV-11, BL-23, KI-3, HT-7, PC-6/ 6 days a week for 4 weeks, 30 min)	(b) Drugs (n = 25) (Diazepam 5.0 mg once a day for 4 weeks)	(1) PSQI	(1) Significant differences in PSQI scores (*P* < 0.05)
Li 2007 [8]	64	(a) 69.8 ± 7.1 (b) 67.3 ± 8.3	(a) 18/14 (b) 17/15	(a) n.r. (b) n.r.	(a) n.r. (b) n.r.	(a) AT + AA (n = 32) (**AT:** HT-7, SP-6, GV-24, EX-HN1, PC-6, LR-3, KI-3; **AA:** Auricular Shenmen/ 6 days a week for 4 weeks, 20–30 min)	(b) Drugs (n = 32) (Diazepam 2.5 mg or estazolam 1 mg once a day for 4 weeks)	(1) PSQI (2) Efficacy standards of Chinese medicine	(1) Significant differences in PSQI scores (*P* < 0.05) (2) Significant differences in Effective rates (*P* < 0.05)
Liu 2006 [30]	62	(a) 69.9 ± 6.9 (b) 67.5 ± 8.2	(a) 15/17 (b) 15/15	(a) n.r. (b) n.r.	(a) n.r. (b) n.r.	(a) AT (n = 32) (HT-7, SP-6, GV-24, EX-HN1, PC-6, LR-3, KI-3, ST-36/ 5 days a week for 4 weeks, 30 min	(b) Drugs (n = 30) (Diazepam 2.5 mg or estazolam 1 mg once a day for 4 weeks)	(1) PSQI	(1) Significant differences in PSQI scores (*P* < 0.05)
Kim 2004 [21]	30	(a) 65.1 ± 9.0 (b) 68.3 ± 10.4	(a) 8/7 (b) 9/6	(a) ISI 21.9 ± 2.0, AIS 17.1 ± 1.6 (b) ISI 22.3 ± 2.1, AIS 17.7 ± 2.5	(a) n.r. (b) n.r.	(a) IA (n = 15) (He7, EH6/ once a day for 3 days)	(b) Sham AT (n = 15)	(1) ISI (2) AIS	(1) Significant differences in ISI scores (*P* < 0.01) (2) Significant differences in AIS scores (*P* < 0.01)
Wang 2004 [31]	64	(a) 42.5 ~ 70.5 (b) 41 ~ 70	(a) 22/12 (b) 17/13	(a) n.r. (b) n.r.	(a) n.r. (b) n.r.	(a) EA + AA (n = 34) (**EA:** HT-7, PC-6, CV-12, ST-36, KI-3; **AA:** Auricular Shenmen/ 5 times a week for 4 weeks, 20–30 min, 40 Hz)	(b) Drugs (n = 30) (Diazepam 2.5 mg or clozapine 25 mg once a day for 4 weeks)	(1) PSQI (2) Efficacy standards of Chinese medicine	(1) Significant differences in PSQI scores (*P* < 0.01) (2) Significant differences in Effective rates (*P* < 0.01)

*Notes. AA* auricular acupuncture, *AIS* Athens insomnia sale, *AT* acupuncture therapy, *EA* electro-acupuncture, *IA* intradermal acupuncture, *ISI* insomnia severity index, *n.r* not reported, *PSQI* Pittsburgh sleep quality index. Adverse effects were not reported for any study

### Study quality

The ROB results are shown in Table 2. With regards to random sequence generation and allocation concealment, five studies had a low ROB [27–31] and eight studies had an unclear ROB [8, 20–26]. With regards to blinding of patients and outcome assessors, two studies had a low ROB [20, 21], and 11 studies had an unclear ROB [8, 22–31]. All RCTs had a low ROB in incomplete outcome data and selective outcome reporting [8, 20–31]. All RCTs had an unclear ROB in other sources of bias [8, 20–31].

**Table 2:** Risk of bias of the studies included in the present review

	Ye 2013 [22]	Li 2012 [23]	Huang 2012 [24]	Wu 2012 [25]	Huang 2011 [26]	Sun 2011 [27]	Ye 2010 [28]	Lee 2009 [20]	Lu 2008 [29]	Li 2007 [8]	Liu 2006 [30]	Kim 2004 [21]	Wang 2004 [31]
1. Was the method of randomization adequate?	U	U	U	U	U	L	L	U	L	U	L	U	L
2. Was the treatment allocation concealed?	U	U	U	U	U	L	L	U	L	U	L	U	L
3. Was the patient blinded to the intervention?	U	U	U	U	U	U	U	L	U	U	U	L	U
4. Were the personnel blinded to the intervention?	U	U	U	U	U	U	U	U	U	U	U	U	U
5. Was the outcome assessor blinded to the intervention?	U	U	U	U	U	U	U	L	U	U	U	L	U
6. Were incomplete outcome data adequately addressed?	L	L	L	L	L	L	L	L	L	L	L	L	L
7. Are reports of the study free of suggestion of selective outcome reporting?	L	L	L	L	L	L	L	L	L	L	L	L	L
8. Was the study apparently free of other problems that could put it at a high risk of bias?	U	U	U	U	U	U	U	U	U	U	U	U	U

*Notes*. Based on the risk of bias assessment tool from the Cochrane handbook for systematic reviews of interventions, high risk of bias: H, low risk of bias: L, uncertain risk of bias: U

### Descriptions of acupuncture treatment

**Fig. 2:**
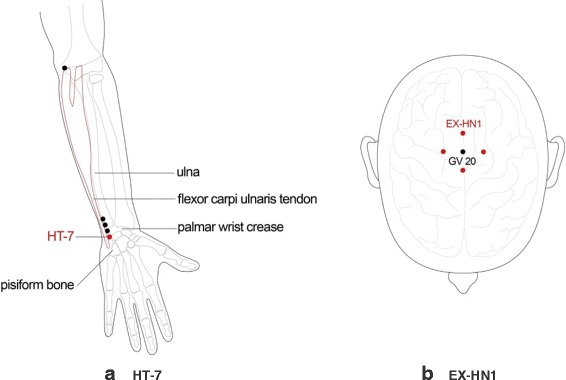
Location of Shenmen (HT-7) and Sishenchong (EX-HN1) acupoints. HT-7 is located in the depression radial to the proximal border of the pisiform bone on the palmar wrist crease. EX-HN1 is a group of four acupoints on the vertex of the head located 1 cun posterior, anterior and lateral to GV 20

The majority of the included RCTs stated that the rationale for acupuncture point selection was drawn from Traditional Chinese Medicine theory. Two studies used intradermal acupuncture [20, 21], five used acupuncture alone [22, 24, 28–30], two used acupuncture and auricular acupuncture [8, 27], two used acupuncture and drugs [23, 26], one used auricular acupuncture [25], and one used electroacupuncture and auricular acupuncture [31]. A total of 33 acupuncture points (24 meridian points and nine auricular acupuncture points) were used for the treatment of insomnia. Acupoints used for insomnia treatment in most trials were Shenmen (HT-7) and Sishencong (EX-HN1) (Fig. 2). The number of acupoints used in each study ranged from two to 11.

### Effects of acupuncture treatment according to PSQI assessment scales

**Fig. 3:**
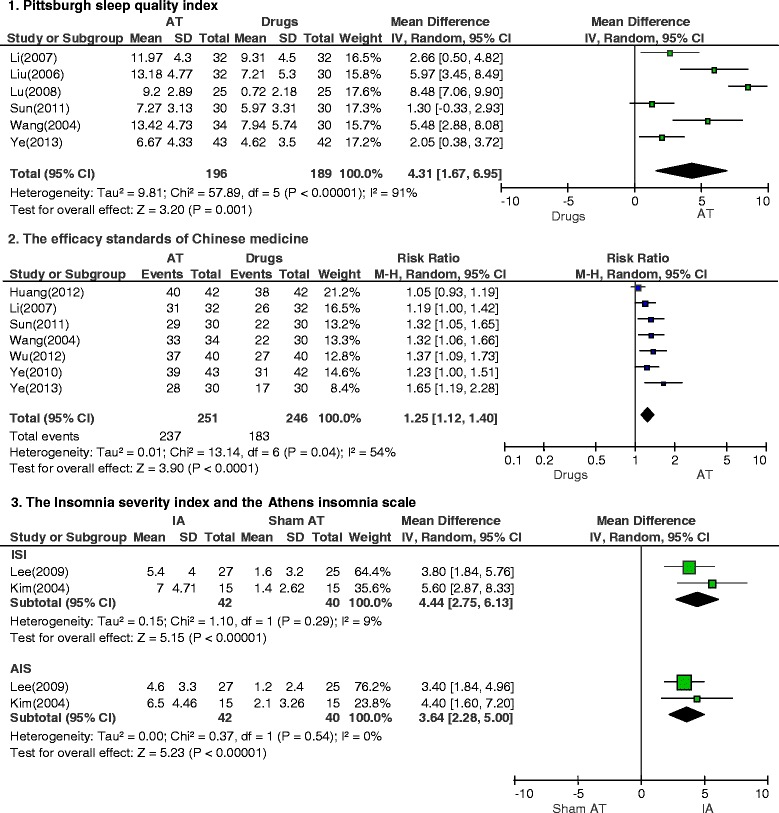
Meta-analysis of acupuncture for insomnia after stroke according to different assessment tools

We conducted a meta-analysis of the study results based on the insomnia assessment scales used (Fig. 3). In six studies that used the PSQI to assess treatment results, acupuncture appeared to be more effective than drugs for treatment of insomnia after stroke (weighted mean difference, 4.31; 95 % CI, 1.67–6.95; *P* = 0.001; *n* = 385, *I*^*2*^ = 91 %).

### Effects of acupuncture treatment according to the efficacy standards of Chinese medicine

In seven studies that used the efficacy standards of Chinese medicine to compare the effects of acupuncture with those of drugs, acupuncture was observed to have a significant difference in reducing insomnia after stroke (RR, 1.25; 95 % CI, 1.12–1.40; *P* < 0.001; n = 497, *I*^*2*^ = 54 %).

### Effects of intradermal acupuncture according to ISI or AIS assessment scales

Studies comparing the effects of intradermal acupuncture with those of sham acupuncture used the ISI or the AIS. In these studies, intradermal acupuncture had a significant difference on insomnia after stroke, as assessed both by the ISI (weighted mean difference, 4.44; 95 % CI, 2.75–6.13; *P* < 0.001; n = 82, *I*^*2*^ = 9 %) and by the AIS (weighted mean difference, 3.64; 95 % CI, 2.28–5.00; *P* < 0.001; n = 82, *I*^*2*^ = 0 %).

### Publication bias

We assessed the publication bias using a funnel plot. However, it was difficult to determine any pattern indicative of publication bias based on the funnel plot’s symmetry owing to the small sample size (fewer than 10 studies, Additional file 1: Figure S1).

## Discussion

The present review suggested that compared to drug treatment, acupuncture might be an effective treatment for insomnia after stroke, and that compared to sham treatment, intradermal acupuncture might have significant effects on insomnia after stroke.

Insomnia is a common complication following stroke, often interfering with activity, recovery, and rehabilitation [32]. Sleep problems have both immediate and long-term health effects. The immediate effects of sleep disturbances include well-being, daytime sleepiness, fatigue, and impaired performance, with their resulting impact on safety [33]. Long-term health effects include hypertension, inflammation, obesity, and glucose intolerance. These long-term effects can lead to chronic diseases and premature death [34]. Additionally, a strong relationship has been found between sleep disturbances and cognitive functioning, regulation of emotions, social problems, and substance abuse [35].

Zhao [12] documented that the clinical efficacy of acupuncture appeared to be supported by evidence obtained from basic neuroendocrinological studies. The evidence has suggested that the clinical efficacy of acupuncture in the treatment of insomnia is potentially mediated by a variety of neurotransmitters, including norepinephrine, melatonin, gamma-aminobutyric acid, and beta-endorphin. Huang [14] reviewed not only clinical trials but also case series and demonstrated that acupuncture is potentially beneficial for the treatment of insomnia. Cheuk [15] reported that acupuncture, when used as an adjunct to other treatments, improved sleep quality as compared with other treatments used alone in a population of patients with diverse medical conditions.

The current review offered significant perspectives. First, we aimed to identify all studies on this topic. There were no restrictions on the review publication language, and a large number of databases were searched. We are therefore confident that our search strategy located all relevant data on the subject. Second, the outcome measures including the PSQI, the efficacy standards of Chinese medicine, the ISI, and the AIS were widely used in practice for the measurements of sleep quality [36]. Significant differences were found between acupuncture treatment and drugs or sham treatment in all of the included assessment tools.

This review also had certain limitations. The scarcity of studies and the methodologically low to moderate quality of the primary data preclude us from drawing confirmative conclusions. The high *I*^*2*^ values were probably because of substantial clinical and methodological variations. Most of the included studies had an unclear risk of bias for blinding, random sequence generation, and allocation concealment; therefore, a preponderance of positive results was observed. Although blinding of the therapists who perform acupuncture would be difficult, blinding of patients, other care providers, and outcome assessors should be attempted in order to minimize the performance and assessment bias of trials.

Therefore, we recommended that any future trial evaluating the effectiveness of acupuncture should have a well-designed protocol in place prior to the trial’s initiation, which is appropriate to properly answer the research questions. Of the many important aspects of improving the quality of trial design, it is critical for any future studies to provide sufficient information about blinding, random sequence generation, and allocation concealment in order to clarify the risk of bias.

Future trials should address the methodological issues through rigorous trial designs, reasonable appraisals, and critical analyses to allow more robust conclusions regarding each treatment’s effectiveness for relieving insomnia after stroke. Future researchers should follow not only the basic guidelines for reporting clinical trials, such as the CONSORT statement, but also the STRICTA recommendations, which provide specific guidelines for reporting acupuncture trials [37, 38]. A large-scale study of multicenter trial is recommended. Long-term follow-up studies are needed to determine the efficacy and safety of treatments for insomnia after stroke and to assess their long-term effects. Moreover, a cost analysis should be considered.

## Conclusions

The results of this study suggested that acupuncture could be effective in relieving insomnia after stroke. Further studies using large samples and a rigorous study design are needed to confirm the role of acupuncture in the treatment of insomnia after stroke.

## Abbreviations

AIS, athens insomnia scale; CI, confidence interval; ISI, insomnia severity index; PSQI, Pittsburgh Sleep Quality Index; RCTs, randomized controlled trials; ROB, risk of bias; RR, risk ratio

## Appendix

### Search strings used for databases

MEDLINE

1. “stroke” OR “apoplexy” OR “cva” OR “cerebrovascular attack” OR “cerebrovascular accident” OR “cerebral infarction” OR “cerebral hemorrhage” 239,345
2. “acupunct” OR “acupress” OR “acupoints” OR “electroacupunct” OR “electro-acupunct” OR “auriculotherapy” OR “auriculoacupunct” 21,349
3. “insomnia” OR “sleep initiation and maintenance disorder” OR “sleep” OR “wakefulness” 142,430
4. 1 AND 2 AND 3 18

EMBASE

1. ‘stroke’/exp OR stroke OR ‘apoplexy’/exp OR apoplexy OR ‘cva’/exp OR cva OR cerebrovascular AND attack OR cerebrovascular AND (‘accident’ /exp OR accident) OR cerebral AND (‘infarction’/exp OR infarction) OR cerebral AND (‘hemorrhage’/exp OR hemorrhage) 42,950
2. acupunct OR acupress OR acupoints OR electroacupunct OR ‘electro acupunct’ OR ‘auriculotherapy’ /exp OR auriculotherapy OR auriculoacupunct 36072
3. insomnia OR sleep AND initiation AND maintenance AND disorder OR sleep OR wakefulness 248,699
4. #1 AND #2 AND #3 7

Cochrane

1. stroke OR apoplexy OR cva OR cerebrovascular attack OR cerebrovascular accident OR cerebral infarction OR cerebral hemorrhage 36368
2. acupunct or acupress or acupoints or electroacupunct or electro-acupunct or auriculotherapy or auriculoacupunct 9101
3. insomnia or sleep initiation and maintenance disorder or sleep or wakefulness 17865
4. #1 AND #2 AND #3 66

CNKI

1. 脑卒中 AND 针 AND 失眠 4
2. 中风 AND 针 AND 失眠 10
3. 脑出血 AND 针 AND 失眠 1
4. stroke AND acupuncture AND insomnia 36

Korean data bases

RISS

1. stroke AND acupuncture AND insomnia 3
2. 뇌졸중 AND 침 AND 불면 0

NDSL

1. stroke AND acupuncture AND insomnia 16
2. 뇌졸중 AND 침 AND 불면 0

OASIS

1. stroke AND acupuncture AND insomnia 3
2. 뇌졸중 AND 침 AND 불면 1

## Additional file

Additional file 1: Figure S1. The funnel plot analysis for identifying publication bias for the meta-analysis of PSQI and efficacy standards of Chinese medicine. MD, mean difference; RR, risk ratio. (DOCX 23 kb)
