# Supplementary material for: Acupuncture for insomnia after stroke: a systematic review and meta-analysis
Source: BMC Complement Altern Med. 2016 Jul 19;16:228. doi: 10.1186/s12906-016-1220-z (PMC4950252; doi:10.1186/s12906-016-1220-z)
Supplement: Additional file 1: Figure S1. — The funnel plot analysis for identifying publication bias for the meta-analysis of PSQI and efficacy standards of Chinese medicine. MD, mean difference; RR, risk ratio. (DOCX 23 kb) [file 12906_2016_1220_MOESM1_ESM.docx]

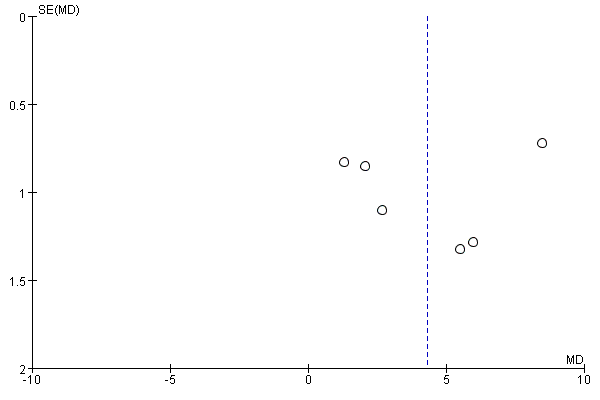

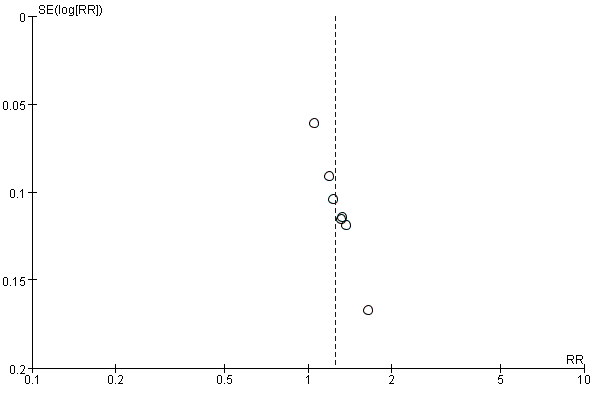


B. Efficacy standards of Chinese medicine

A. PSQI

**Additional file 1: Figure S1.** The funnel plot analysis for identifying publication bias for the meta-analysis of PSQI and efficacy standards of Chinese medicine. MD, mean difference; RR, risk ratio.
